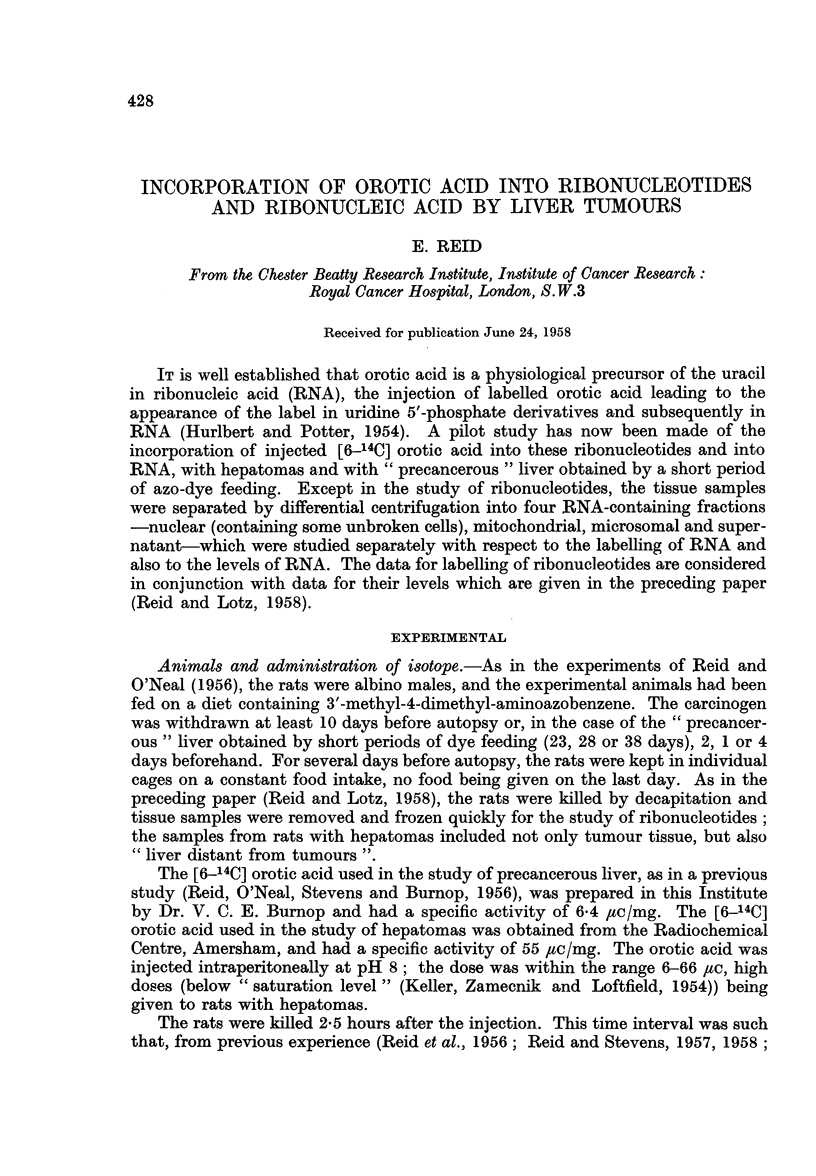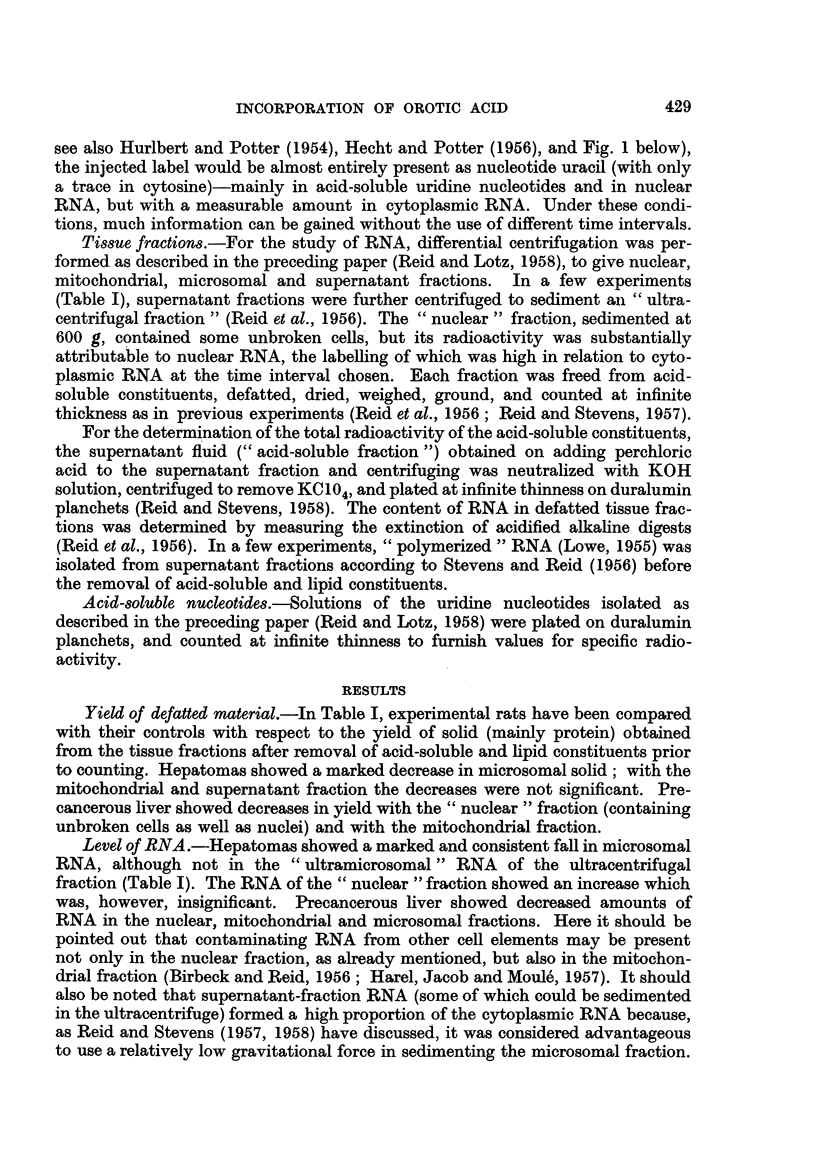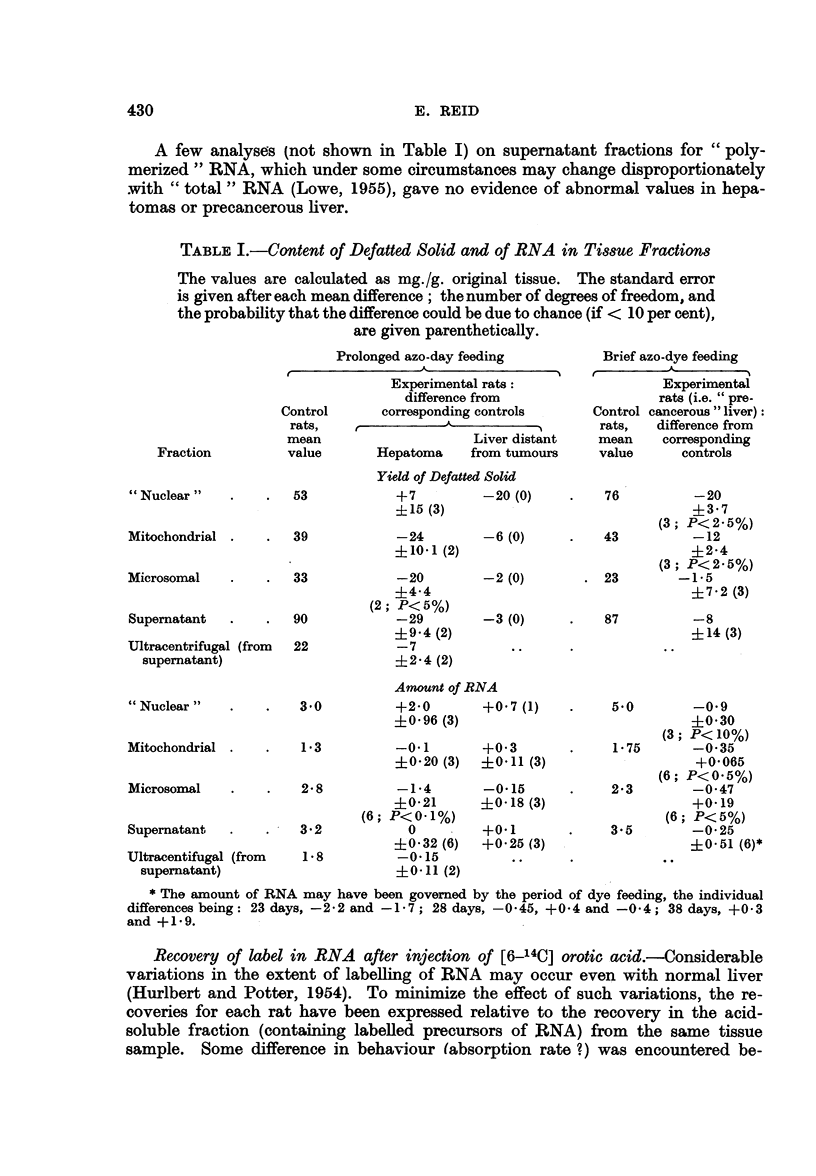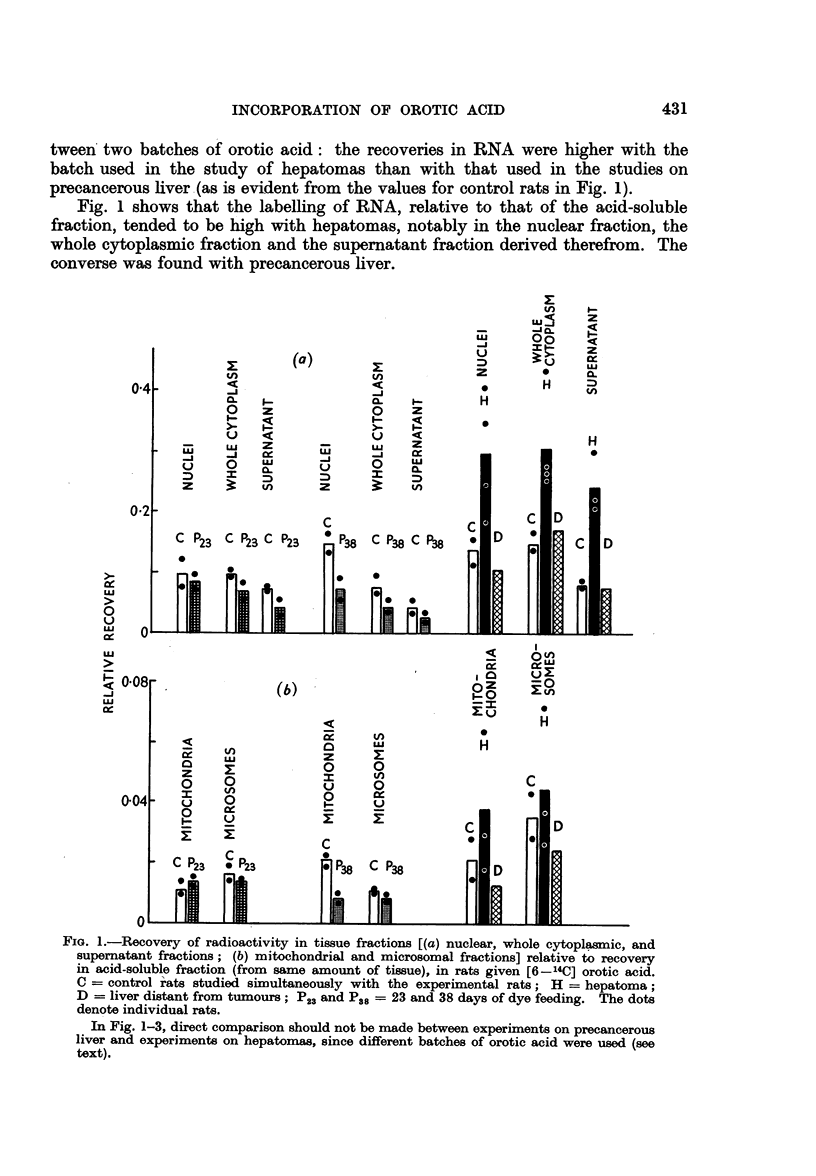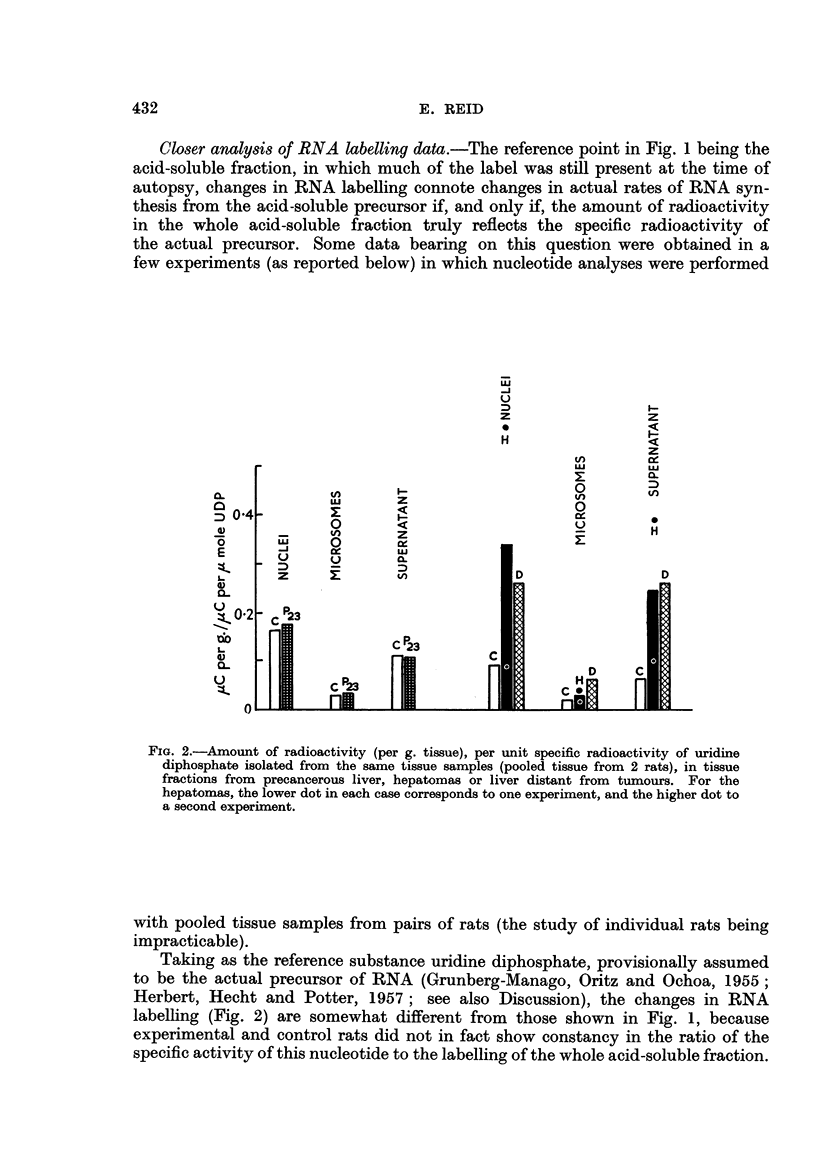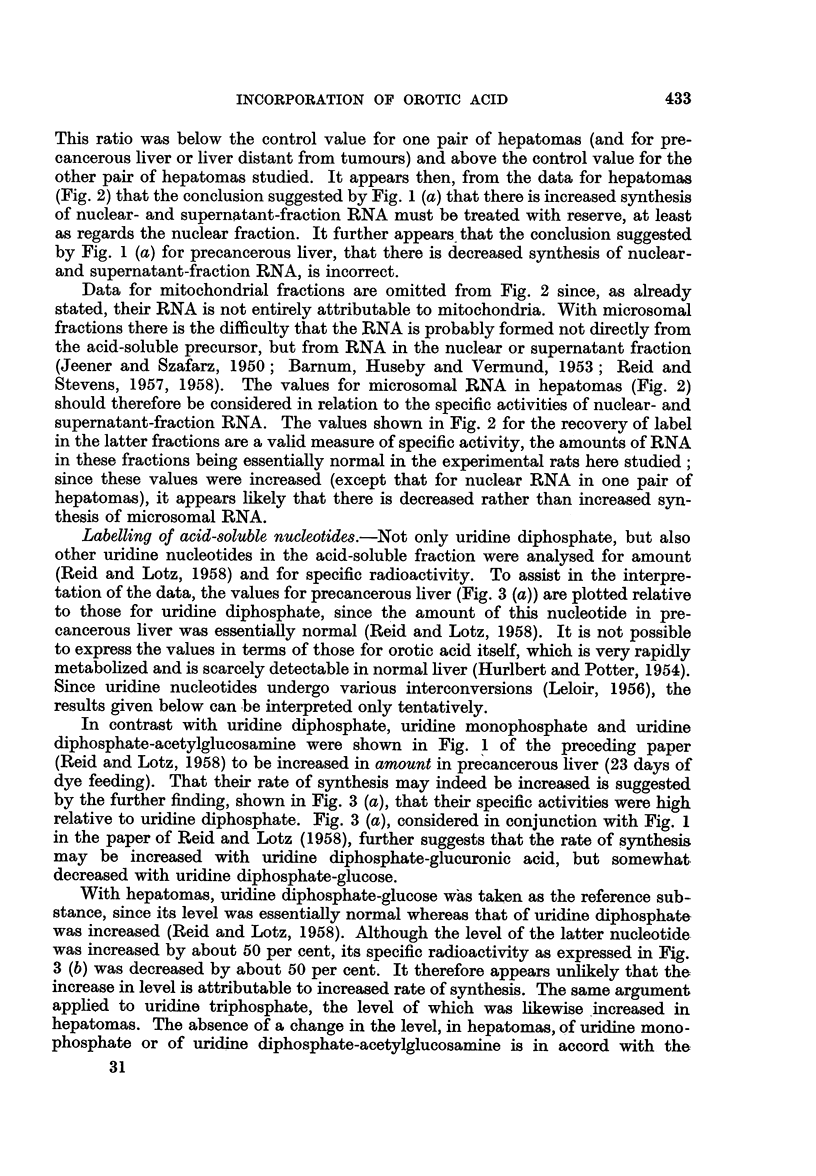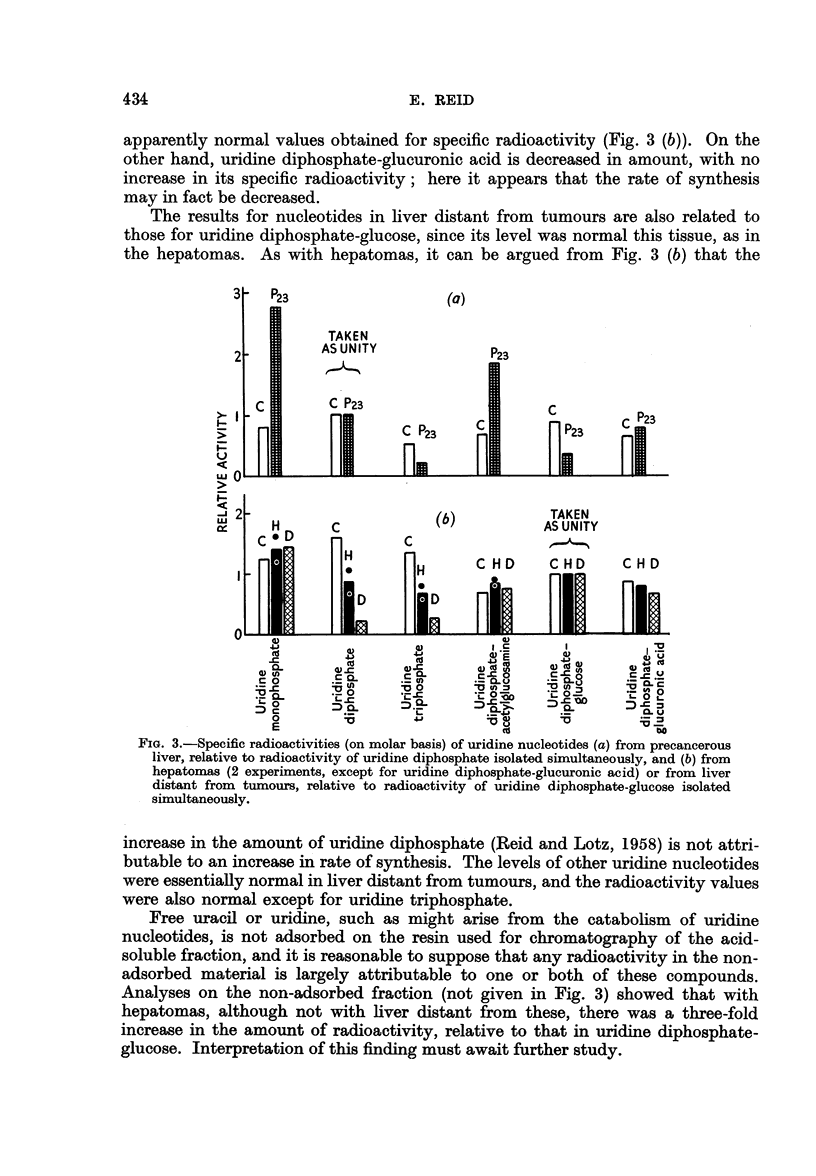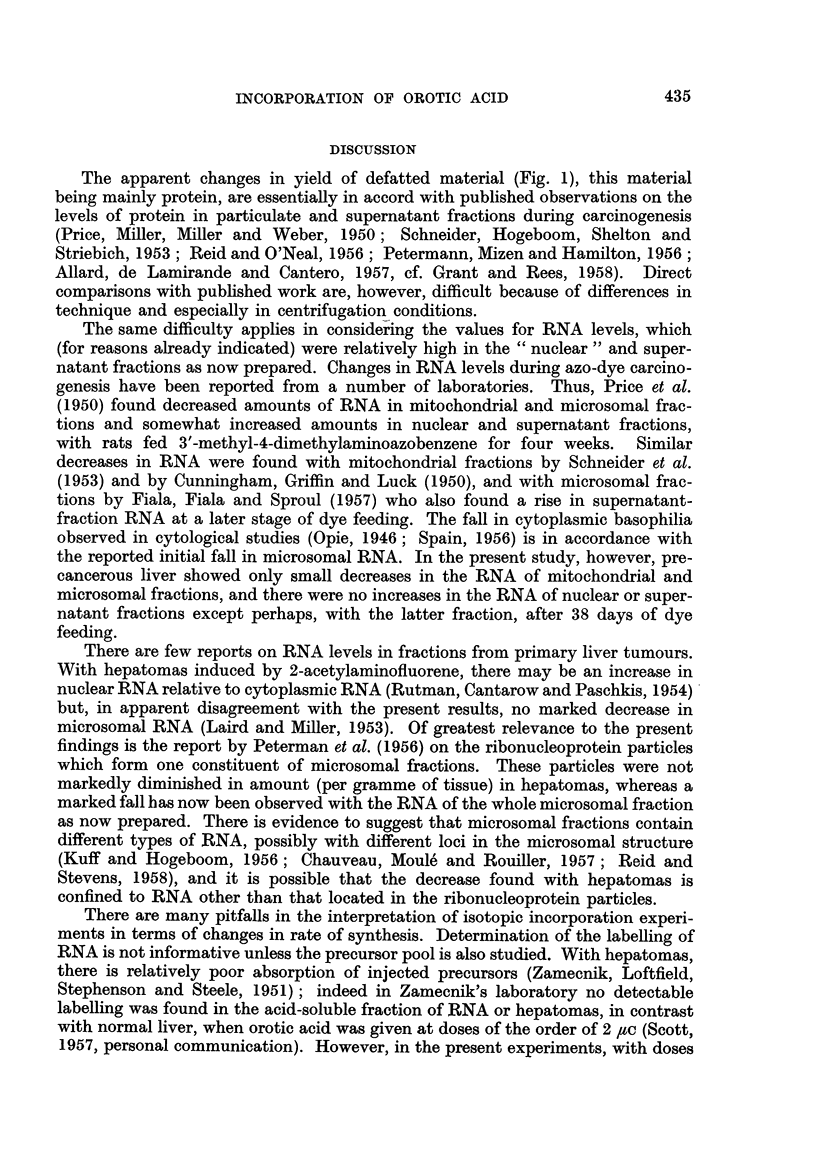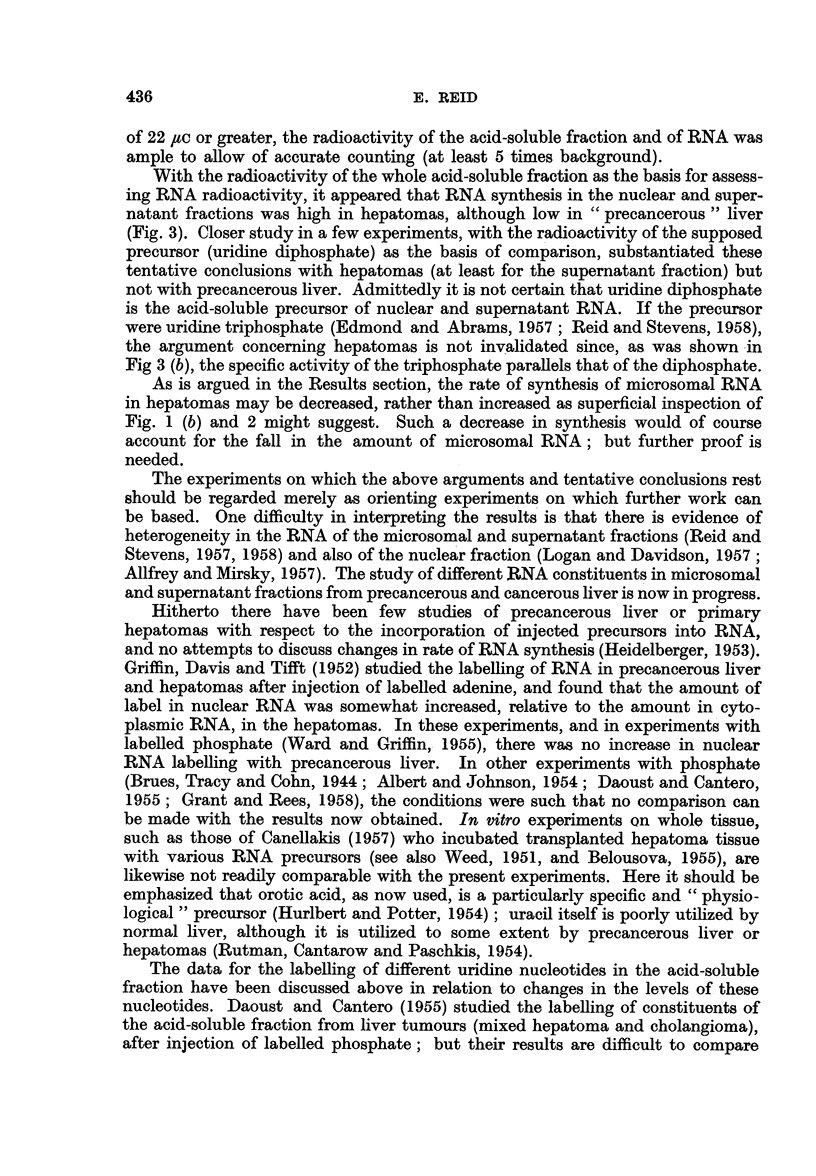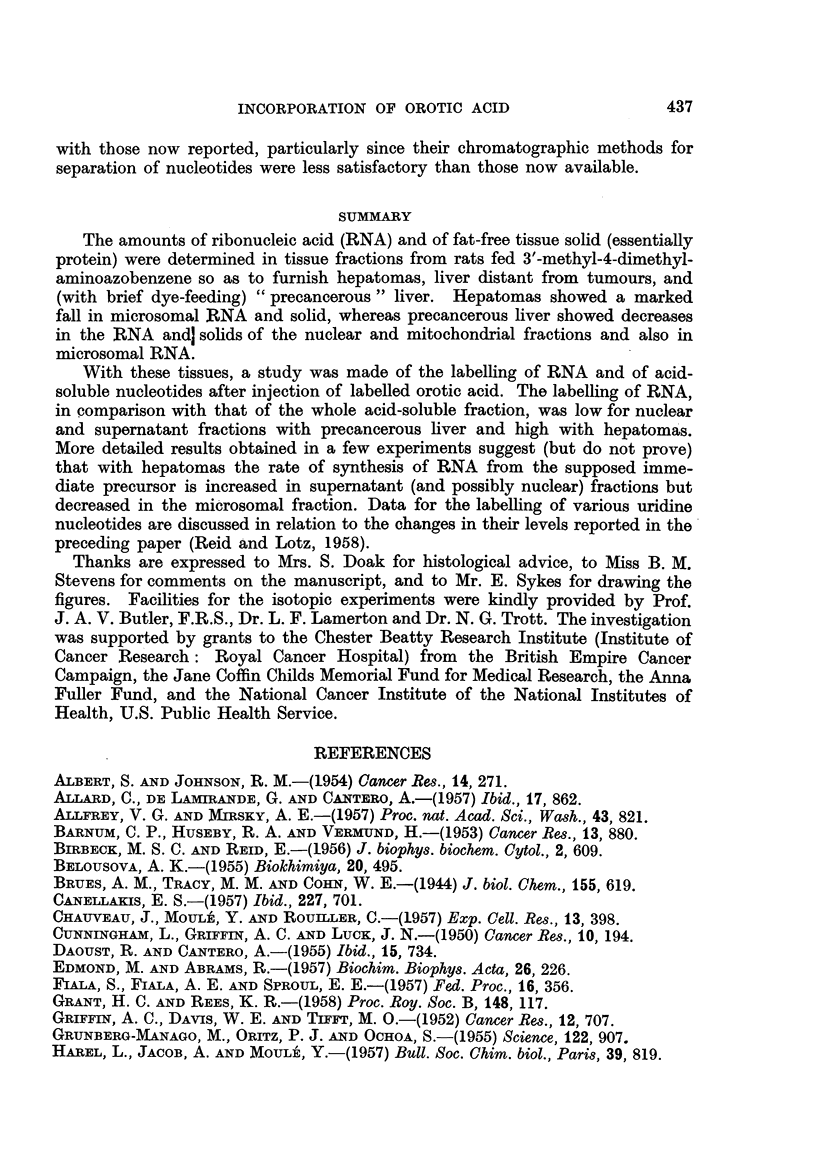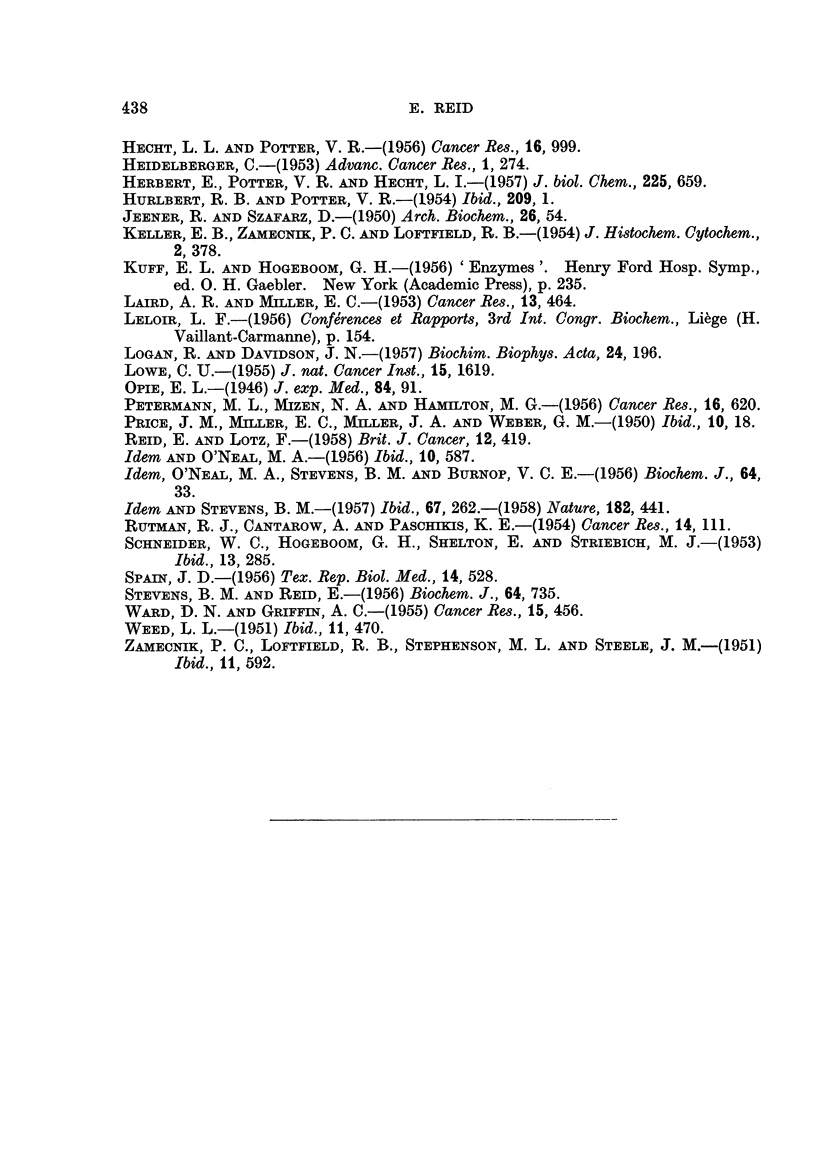# Incorporation of Orotic Acid into Ribonucleotides and Ribonucleic Acid by Liver Tumours

**DOI:** 10.1038/bjc.1958.51

**Published:** 1958-09

**Authors:** E. Reid


### INCORPORATION OF OROTIC ACID INTO RIBONUCLEOTIDES AND RIBONUCLEIC ACID BY LIVER TUMOURS

E. REID

*From the Chester Beatty Research Institute, Institute of Cancer Research : Royal Cancer Hospital, London, S.W.3*

Received for publication June 24, 1958

IT is well established that orotic acid is a physiological precursor of the uracil in ribonucleic acid (RNA), the injection of labelled orotic acid leading to the appearance of the label in uridine 5′-phosphate derivatives and subsequently in RNA (Hurlbert and Potter, 1954). A pilot study has now been made of the incorporation of injected [6–$^{14}$C] orotic acid into these ribonucleotides and into RNA, with hepatomas and with " precancerous " liver obtained by a short period of azo-dye feeding. Except in the study of ribonucleotides, the tissue samples were separated by differential centrifugation into four RNA-containing fractions —nuclear (containing some unbroken cells), mitochondrial, microsomal and supernatant—which were studied separately with respect to the labelling of RNA and also to the levels of RNA. The data for labelling of ribonucleotides are considered in conjunction with data for their levels which are given in the preceding paper (Reid and Lotz, 1958).

### EXPERIMENTAL

*Animals and administration of isotope.*—As in the experiments of Reid and O'Neal (1956), the rats were albino males, and the experimental animals had been fed on a diet containing 3′-methyl-4-dimethyl-aminoazobenzene. The carcinogen was withdrawn at least 10 days before autopsy or, in the case of the " precancerous " liver obtained by short periods of dye feeding (23, 28 or 38 days), 2, 1 or 4 days beforehand. For several days before autopsy, the rats were kept in individual cages on a constant food intake, no food being given on the last day. As in the preceding paper (Reid and Lotz, 1958), the rats were killed by decapitation and tissue samples were removed and frozen quickly for the study of ribonucleotides ; the samples from rats with hepatomas included not only tumour tissue, but also " liver distant from tumours ".

The [6–$^{14}$C] orotic acid used in the study of precancerous liver, as in a previous study (Reid, O'Neal, Stevens and Burnop, 1956), was prepared in this Institute by Dr. V. C. E. Burnop and had a specific activity of 6·4 $\mu$c/mg. The [6–$^{14}$C] orotic acid used in the study of hepatomas was obtained from the Radiochemical Centre, Amersham, and had a specific activity of 55 $\mu$c/mg. The orotic acid was injected intraperitoneally at pH 8 ; the dose was within the range 6–66 $\mu$c, high doses (below " saturation level " (Keller, Zamecnik and Loftfield, 1954)) being given to rats with hepatomas.

The rats were killed 2·5 hours after the injection. This time interval was such that, from previous experience (Reid *et al.*, 1956 ; Reid and Stevens, 1957, 1958 ; see also Hurlbert and Potter (1954), Hecht and Potter (1956), and Fig. 1 below), the injected label would be almost entirely present as nucleotide uracil (with only a trace in cytosine)—mainly in acid-soluble uridine nucleotides and in nuclear RNA, but with a measurable amount in cytoplasmic RNA. Under these conditions, much information can be gained without the use of different time intervals.

*Tissue fractions.*—For the study of RNA, differential centrifugation was performed as described in the preceding paper (Reid and Lotz, 1958), to give nuclear, mitochondrial, microsomal and supernatant fractions. In a few experiments (Table I), supernatant fractions were further centrifuged to sediment an " ultracentrifugal fraction " (Reid *et al.*, 1956). The " nuclear " fraction, sedimented at 600 *g*, contained some unbroken cells, but its radioactivity was substantially attributable to nuclear RNA, the labelling of which was high in relation to cytoplasmic RNA at the time interval chosen. Each fraction was freed from acid-soluble constituents, defatted, dried, weighed, ground, and counted at infinite thickness as in previous experiments (Reid *et al.*, 1956 ; Reid and Stevens, 1957).

For the determination of the total radioactivity of the acid-soluble constituents, the supernatant fluid (" acid-soluble fraction ") obtained on adding perchloric acid to the supernatant fraction and centrifuging was neutralized with KOH solution, centrifuged to remove $KClO_4$, and plated at infinite thinness on duralumin planchets (Reid and Stevens, 1958). The content of RNA in defatted tissue fractions was determined by measuring the extinction of acidified alkaline digests (Reid *et al.*, 1956). In a few experiments, " polymerized " RNA (Lowe, 1955) was isolated from supernatant fractions according to Stevens and Reid (1956) before the removal of acid-soluble and lipid constituents.

*Acid-soluble nucleotides.*—Solutions of the uridine nucleotides isolated as described in the preceding paper (Reid and Lotz, 1958) were plated on duralumin planchets, and counted at infinite thinness to furnish values for specific radioactivity.

### RESULTS

*Yield of defatted material.*—In Table I, experimental rats have been compared with their controls with respect to the yield of solid (mainly protein) obtained from the tissue fractions after removal of acid-soluble and lipid constituents prior to counting. Hepatomas showed a marked decrease in microsomal solid ; with the mitochondrial and supernatant fraction the decreases were not significant. Precancerous liver showed decreases in yield with the " nuclear " fraction (containing unbroken cells as well as nuclei) and with the mitochondrial fraction.

*Level of RNA.*—Hepatomas showed a marked and consistent fall in microsomal RNA, although not in the " ultramicrosomal " RNA of the ultracentrifugal fraction (Table I). The RNA of the " nuclear " fraction showed an increase which was, however, insignificant. Precancerous liver showed decreased amounts of RNA in the nuclear, mitochondrial and microsomal fractions. Here it should be pointed out that contaminating RNA from other cell elements may be present not only in the nuclear fraction, as already mentioned, but also in the mitochondrial fraction (Birbeck and Reid, 1956 ; Harel, Jacob and Moulé, 1957). It should also be noted that supernatant-fraction RNA (some of which could be sedimented in the ultracentrifuge) formed a high proportion of the cytoplasmic RNA because, as Reid and Stevens (1957, 1958) have discussed, it was considered advantageous to use a relatively low gravitational force in sedimenting the microsomal fraction.

A few analyses (not shown in Table I) on supernatant fractions for " polymerized " RNA, which under some circumstances may change disproportionately with " total " RNA (Lowe, 1955), gave no evidence of abnormal values in hepatomas or precancerous liver.

TABLE I.—*Content of Defatted Solid and of RNA in Tissue Fractions*

The values are calculated as mg./g. original tissue. The standard error is given after each mean difference ; the number of degrees of freedom, and the probability that the difference could be due to chance (if < 10 per cent), are given parenthetically.

| | Prolonged azo-day feeding | | | Brief azo-dye feeding | |
|---|---|---|---|---|---|
| | | Experimental rats : difference from corresponding controls | | | |
| Fraction | Control rats, mean value | Hepatoma | Liver distant from tumours | Control rats, mean value | Experimental rats (i.e. " precancerous " liver) : difference from corresponding controls |
| | | *Yield of Defatted Solid* | | | |
| " Nuclear " | 53 | +7 ±15 (3) | −20 (0) | 76 | −20 ±3·7 (3 ; $P<2·5\%$) |
| Mitochondrial | 39 | −24 ±10·1 (2) | −6 (0) | 43 | −12 ±2·4 (3 ; $P<2·5\%$) |
| Microsomal | 33 | −20 ±4·4 (2 ; $P<5\%$) | −2 (0) | 23 | −1·5 ±7·2 (3) |
| Supernatant | 90 | −29 ±9·4 (2) | −3 (0) | 87 | −8 ±14 (3) |
| Ultracentrifugal (from supernatant) | 22 | −7 ±2·4 (2) | .. | | .. |
| | | *Amount of RNA* | | | |
| " Nuclear " | 3·0 | +2·0 ±0·96 (3) | +0·7 (1) | 5·0 | −0·9 ±0·30 (3 ; $P<10\%$) |
| Mitochondrial | 1·3 | −0·1 ±0·20 (3) | +0·3 ±0·11 (3) | 1·75 | −0·35 +0·065 (6 ; $P<0·5\%$) |
| Microsomal | 2·8 | −1·4 ±0·21 (6 ; $P<0·1\%$) | −0·15 ±0·18 (3) | 2·3 | −0·47 +0·19 (6 ; $P<5\%$) |
| Supernatant | 3·2 | 0 ±0·32 (6) | +0·1 +0·25 (3) | 3·5 | −0·25 ±0·51 (6)* |
| Ultracentifugal (from supernatant) | 1·8 | −0·15 ±0·11 (2) | .. | | .. |

* The amount of RNA may have been governed by the period of dye feeding, the individual differences being : 23 days, −2·2 and −1·7 ; 28 days, −0·45, +0·4 and −0·4 ; 38 days, +0·3 and +1·9.

*Recovery of label in RNA after injection of* [6–$^{14}$C] *orotic acid.*—Considerable variations in the extent of labelling of RNA may occur even with normal liver (Hurlbert and Potter, 1954). To minimize the effect of such variations, the recoveries for each rat have been expressed relative to the recovery in the acid-soluble fraction (containing labelled precursors of RNA) from the same tissue sample. Some difference in behaviour (absorption rate ?) was encountered between two batches of orotic acid: the recoveries in RNA were higher with the batch used in the study of hepatomas than with that used in the studies on precancerous liver (as is evident from the values for control rats in Fig. 1).

Fig. 1 shows that the labelling of RNA, relative to that of the acid-soluble fraction, tended to be high with hepatomas, notably in the nuclear fraction, the whole cytoplasmic fraction and the supernatant fraction derived therefrom. The converse was found with precancerous liver.

FIG. 1.—Recovery of radioactivity in tissue fractions [(*a*) nuclear, whole cytoplasmic, and supernatant fractions; (*b*) mitochondrial and microsomal fractions] relative to recovery in acid-soluble fraction (from same amount of tissue), in rats given [6—$^{14}$C] orotic acid. C = control rats studied simultaneously with the experimental rats; H = hepatoma; D = liver distant from tumours; $P_{23}$ and $P_{38}$ = 23 and 38 days of dye feeding. The dots denote individual rats.

In Fig. 1–3, direct comparison should not be made between experiments on precancerous liver and experiments on hepatomas, since different batches of orotic acid were used (see text).

*Closer analysis of RNA labelling data.*—The reference point in Fig. 1 being the acid-soluble fraction, in which much of the label was still present at the time of autopsy, changes in RNA labelling connote changes in actual rates of RNA synthesis from the acid-soluble precursor if, and only if, the amount of radioactivity in the whole acid-soluble fraction truly reflects the specific radioactivity of the actual precursor. Some data bearing on this question were obtained in a few experiments (as reported below) in which nucleotide analyses were performed

FIG. 2.—Amount of radioactivity (per g. tissue), per unit specific radioactivity of uridine diphosphate isolated from the same tissue samples (pooled tissue from 2 rats), in tissue fractions from precancerous liver, hepatomas or liver distant from tumours. For the hepatomas, the lower dot in each case corresponds to one experiment, and the higher dot to a second experiment.

with pooled tissue samples from pairs of rats (the study of individual rats being impracticable).

Taking as the reference substance uridine diphosphate, provisionally assumed to be the actual precursor of RNA (Grunberg-Manago, Oritz and Ochoa, 1955 ; Herbert, Hecht and Potter, 1957 ; see also Discussion), the changes in RNA labelling (Fig. 2) are somewhat different from those shown in Fig. 1, because experimental and control rats did not in fact show constancy in the ratio of the specific activity of this nucleotide to the labelling of the whole acid-soluble fraction.

This ratio was below the control value for one pair of hepatomas (and for precancerous liver or liver distant from tumours) and above the control value for the other pair of hepatomas studied. It appears then, from the data for hepatomas (Fig. 2) that the conclusion suggested by Fig. 1 (*a*) that there is increased synthesis of nuclear- and supernatant-fraction RNA must be treated with reserve, at least as regards the nuclear fraction. It further appears that the conclusion suggested by Fig. 1 (*a*) for precancerous liver, that there is decreased synthesis of nuclear- and supernatant-fraction RNA, is incorrect.

Data for mitochondrial fractions are omitted from Fig. 2 since, as already stated, their RNA is not entirely attributable to mitochondria. With microsomal fractions there is the difficulty that the RNA is probably formed not directly from the acid-soluble precursor, but from RNA in the nuclear or supernatant fraction (Jeener and Szafarz, 1950; Barnum, Huseby and Vermund, 1953; Reid and Stevens, 1957, 1958). The values for microsomal RNA in hepatomas (Fig. 2) should therefore be considered in relation to the specific activities of nuclear- and supernatant-fraction RNA. The values shown in Fig. 2 for the recovery of label in the latter fractions are a valid measure of specific activity, the amounts of RNA in these fractions being essentially normal in the experimental rats here studied; since these values were increased (except that for nuclear RNA in one pair of hepatomas), it appears likely that there is decreased rather than increased synthesis of microsomal RNA.

*Labelling of acid-soluble nucleotides.*—Not only uridine diphosphate, but also other uridine nucleotides in the acid-soluble fraction were analysed for amount (Reid and Lotz, 1958) and for specific radioactivity. To assist in the interpretation of the data, the values for precancerous liver (Fig. 3 (*a*)) are plotted relative to those for uridine diphosphate, since the amount of this nucleotide in precancerous liver was essentially normal (Reid and Lotz, 1958). It is not possible to express the values in terms of those for orotic acid itself, which is very rapidly metabolized and is scarcely detectable in normal liver (Hurlbert and Potter, 1954). Since uridine nucleotides undergo various interconversions (Leloir, 1956), the results given below can be interpreted only tentatively.

In contrast with uridine diphosphate, uridine monophosphate and uridine diphosphate-acetylglucosamine were shown in Fig. 1 of the preceding paper (Reid and Lotz, 1958) to be increased in *amount* in precancerous liver (23 days of dye feeding). That their rate of synthesis may indeed be increased is suggested by the further finding, shown in Fig. 3 (*a*), that their specific activities were high relative to uridine diphosphate. Fig. 3 (*a*), considered in conjunction with Fig. 1 in the paper of Reid and Lotz (1958), further suggests that the rate of synthesis may be increased with uridine diphosphate-glucuronic acid, but somewhat decreased with uridine diphosphate-glucose.

With hepatomas, uridine diphosphate-glucose was taken as the reference substance, since its level was essentially normal whereas that of uridine diphosphate was increased (Reid and Lotz, 1958). Although the level of the latter nucleotide was increased by about 50 per cent, its specific radioactivity as expressed in Fig. 3 (*b*) was decreased by about 50 per cent. It therefore appears unlikely that the increase in level is attributable to increased rate of synthesis. The same argument applied to uridine triphosphate, the level of which was likewise increased in hepatomas. The absence of a change in the level, in hepatomas, of uridine monophosphate or of uridine diphosphate-acetylglucosamine is in accord with the apparently normal values obtained for specific radioactivity (Fig. 3 (*b*)). On the other hand, uridine diphosphate-glucuronic acid is decreased in amount, with no increase in its specific radioactivity ; here it appears that the rate of synthesis may in fact be decreased.

The results for nucleotides in liver distant from tumours are also related to those for uridine diphosphate-glucose, since its level was normal this tissue, as in the hepatomas. As with hepatomas, it can be argued from Fig. 3 (*b*) that the

FIG. 3.—Specific radioactivities (on molar basis) of uridine nucleotides (*a*) from precancerous liver, relative to radioactivity of uridine diphosphate isolated simultaneously, and (*b*) from hepatomas (2 experiments, except for uridine diphosphate-glucuronic acid) or from liver distant from tumours, relative to radioactivity of uridine diphosphate-glucose isolated simultaneously.

increase in the amount of uridine diphosphate (Reid and Lotz, 1958) is not attributable to an increase in rate of synthesis. The levels of other uridine nucleotides were essentially normal in liver distant from tumours, and the radioactivity values were also normal except for uridine triphosphate.

Free uracil or uridine, such as might arise from the catabolism of uridine nucleotides, is not adsorbed on the resin used for chromatography of the acid-soluble fraction, and it is reasonable to suppose that any radioactivity in the non-adsorbed material is largely attributable to one or both of these compounds. Analyses on the non-adsorbed fraction (not given in Fig. 3) showed that with hepatomas, although not with liver distant from these, there was a three-fold increase in the amount of radioactivity, relative to that in uridine diphosphate-glucose. Interpretation of this finding must await further study.

### DISCUSSION

The apparent changes in yield of defatted material (Fig. 1), this material being mainly protein, are essentially in accord with published observations on the levels of protein in particulate and supernatant fractions during carcinogenesis (Price, Miller, Miller and Weber, 1950; Schneider, Hogeboom, Shelton and Striebich, 1953; Reid and O'Neal, 1956; Petermann, Mizen and Hamilton, 1956; Allard, de Lamirande and Cantero, 1957, cf. Grant and Rees, 1958). Direct comparisons with published work are, however, difficult because of differences in technique and especially in centrifugation conditions.

The same difficulty applies in considering the values for RNA levels, which (for reasons already indicated) were relatively high in the "nuclear" and supernatant fractions as now prepared. Changes in RNA levels during azo-dye carcinogenesis have been reported from a number of laboratories. Thus, Price *et al.* (1950) found decreased amounts of RNA in mitochondrial and microsomal fractions and somewhat increased amounts in nuclear and supernatant fractions, with rats fed 3′-methyl-4-dimethylaminoazobenzene for four weeks. Similar decreases in RNA were found with mitochondrial fractions by Schneider *et al.* (1953) and by Cunningham, Griffin and Luck (1950), and with microsomal fractions by Fiala, Fiala and Sproul (1957) who also found a rise in supernatant-fraction RNA at a later stage of dye feeding. The fall in cytoplasmic basophilia observed in cytological studies (Opie, 1946; Spain, 1956) is in accordance with the reported initial fall in microsomal RNA. In the present study, however, precancerous liver showed only small decreases in the RNA of mitochondrial and microsomal fractions, and there were no increases in the RNA of nuclear or supernatant fractions except perhaps, with the latter fraction, after 38 days of dye feeding.

There are few reports on RNA levels in fractions from primary liver tumours. With hepatomas induced by 2-acetylaminofluorene, there may be an increase in nuclear RNA relative to cytoplasmic RNA (Rutman, Cantarow and Paschkis, 1954) but, in apparent disagreement with the present results, no marked decrease in microsomal RNA (Laird and Miller, 1953). Of greatest relevance to the present findings is the report by Peterman *et al.* (1956) on the ribonucleoprotein particles which form one constituent of microsomal fractions. These particles were not markedly diminished in amount (per gramme of tissue) in hepatomas, whereas a marked fall has now been observed with the RNA of the whole microsomal fraction as now prepared. There is evidence to suggest that microsomal fractions contain different types of RNA, possibly with different loci in the microsomal structure (Kuff and Hogeboom, 1956; Chauveau, Moulé and Rouiller, 1957; Reid and Stevens, 1958), and it is possible that the decrease found with hepatomas is confined to RNA other than that located in the ribonucleoprotein particles.

There are many pitfalls in the interpretation of isotopic incorporation experiments in terms of changes in rate of synthesis. Determination of the labelling of RNA is not informative unless the precursor pool is also studied. With hepatomas, there is relatively poor absorption of injected precursors (Zamecnik, Loftfield, Stephenson and Steele, 1951); indeed in Zamecnik's laboratory no detectable labelling was found in the acid-soluble fraction of RNA or hepatomas, in contrast with normal liver, when orotic acid was given at doses of the order of 2 $\mu$c (Scott, 1957, personal communication). However, in the present experiments, with doses of 22 μc or greater, the radioactivity of the acid-soluble fraction and of RNA was ample to allow of accurate counting (at least 5 times background).

With the radioactivity of the whole acid-soluble fraction as the basis for assessing RNA radioactivity, it appeared that RNA synthesis in the nuclear and supernatant fractions was high in hepatomas, although low in "precancerous" liver (Fig. 3). Closer study in a few experiments, with the radioactivity of the supposed precursor (uridine diphosphate) as the basis of comparison, substantiated these tentative conclusions with hepatomas (at least for the supernatant fraction) but not with precancerous liver. Admittedly it is not certain that uridine diphosphate is the acid-soluble precursor of nuclear and supernatant RNA. If the precursor were uridine triphosphate (Edmond and Abrams, 1957; Reid and Stevens, 1958), the argument concerning hepatomas is not invalidated since, as was shown in Fig 3 (*b*), the specific activity of the triphosphate parallels that of the diphosphate.

As is argued in the Results section, the rate of synthesis of microsomal RNA in hepatomas may be decreased, rather than increased as superficial inspection of Fig. 1 (*b*) and 2 might suggest. Such a decrease in synthesis would of course account for the fall in the amount of microsomal RNA; but further proof is needed.

The experiments on which the above arguments and tentative conclusions rest should be regarded merely as orienting experiments on which further work can be based. One difficulty in interpreting the results is that there is evidence of heterogeneity in the RNA of the microsomal and supernatant fractions (Reid and Stevens, 1957, 1958) and also of the nuclear fraction (Logan and Davidson, 1957; Allfrey and Mirsky, 1957). The study of different RNA constituents in microsomal and supernatant fractions from precancerous and cancerous liver is now in progress.

Hitherto there have been few studies of precancerous liver or primary hepatomas with respect to the incorporation of injected precursors into RNA, and no attempts to discuss changes in rate of RNA synthesis (Heidelberger, 1953). Griffin, Davis and Tifft (1952) studied the labelling of RNA in precancerous liver and hepatomas after injection of labelled adenine, and found that the amount of label in nuclear RNA was somewhat increased, relative to the amount in cytoplasmic RNA, in the hepatomas. In these experiments, and in experiments with labelled phosphate (Ward and Griffin, 1955), there was no increase in nuclear RNA labelling with precancerous liver. In other experiments with phosphate (Brues, Tracy and Cohn, 1944; Albert and Johnson, 1954; Daoust and Cantero, 1955; Grant and Rees, 1958), the conditions were such that no comparison can be made with the results now obtained. *In vitro* experiments on whole tissue, such as those of Canellakis (1957) who incubated transplanted hepatoma tissue with various RNA precursors (see also Weed, 1951, and Belousova, 1955), are likewise not readily comparable with the present experiments. Here it should be emphasized that orotic acid, as now used, is a particularly specific and "physiological" precursor (Hurlbert and Potter, 1954); uracil itself is poorly utilized by normal liver, although it is utilized to some extent by precancerous liver or hepatomas (Rutman, Cantarow and Paschkis, 1954).

The data for the labelling of different uridine nucleotides in the acid-soluble fraction have been discussed above in relation to changes in the levels of these nucleotides. Daoust and Cantero (1955) studied the labelling of constituents of the acid-soluble fraction from liver tumours (mixed hepatoma and cholangioma), after injection of labelled phosphate; but their results are difficult to compare with those now reported, particularly since their chromatographic methods for separation of nucleotides were less satisfactory than those now available.

SUMMARY

The amounts of ribonucleic acid (RNA) and of fat-free tissue solid (essentially protein) were determined in tissue fractions from rats fed 3′-methyl-4-dimethylaminoazobenzene so as to furnish hepatomas, liver distant from tumours, and (with brief dye-feeding) " precancerous " liver. Hepatomas showed a marked fall in microsomal RNA and solid, whereas precancerous liver showed decreases in the RNA and solids of the nuclear and mitochondrial fractions and also in microsomal RNA.

With these tissues, a study was made of the labelling of RNA and of acid-soluble nucleotides after injection of labelled orotic acid. The labelling of RNA, in comparison with that of the whole acid-soluble fraction, was low for nuclear and supernatant fractions with precancerous liver and high with hepatomas. More detailed results obtained in a few experiments suggest (but do not prove) that with hepatomas the rate of synthesis of RNA from the supposed immediate precursor is increased in supernatant (and possibly nuclear) fractions but decreased in the microsomal fraction. Data for the labelling of various uridine nucleotides are discussed in relation to the changes in their levels reported in the preceding paper (Reid and Lotz, 1958).

Thanks are expressed to Mrs. S. Doak for histological advice, to Miss B. M. Stevens for comments on the manuscript, and to Mr. E. Sykes for drawing the figures. Facilities for the isotopic experiments were kindly provided by Prof. J. A. V. Butler, F.R.S., Dr. L. F. Lamerton and Dr. N. G. Trott. The investigation was supported by grants to the Chester Beatty Research Institute (Institute of Cancer Research : Royal Cancer Hospital) from the British Empire Cancer Campaign, the Jane Coffin Childs Memorial Fund for Medical Research, the Anna Fuller Fund, and the National Cancer Institute of the National Institutes of Health, U.S. Public Health Service.

### REFERENCES

Albert, S. and Johnson, R. M.—(1954) *Cancer Res.*, **14**, 271.
Allard, C., de Lamirande, G. and Cantero, A.—(1957) *Ibid.*, **17**, 862.
Allfrey, V. G. and Mirsky, A. E.—(1957) *Proc. nat. Acad. Sci., Wash.*, **43**, 821.
Barnum, C. P., Huseby, R. A. and Vermund, H.—(1953) *Cancer Res.*, **13**, 880.
Birbeck, M. S. C. and Reid, E.—(1956) *J. biophys. biochem. Cytol.*, **2**, 609.
Belousova, A. K.—(1955) *Biokhimiya*, **20**, 495.
Brues, A. M., Tracy, M. M. and Cohn, W. E.—(1944) *J. biol. Chem.*, **155**, 619.
Canellakis, E. S.—(1957) *Ibid.*, **227**, 701.
Chauveau, J., Moulé, Y. and Rouiller, C.—(1957) *Exp. Cell. Res.*, **13**, 398.
Cunningham, L., Griffin, A. C. and Luck, J. N.—(1950) *Cancer Res.*, **10**, 194.
Daoust, R. and Cantero, A.—(1955) *Ibid.*, **15**, 734.
Edmond, M. and Abrams, R.—(1957) *Biochim. Biophys. Acta*, **26**, 226.
Fiala, S., Fiala, A. E. and Sproul, E. E.—(1957) *Fed. Proc.*, **16**, 356.
Grant, H. C. and Rees, K. R.—(1958) *Proc. Roy. Soc.* B, **148**, 117.
Griffin, A. C., Davis, W. E. and Tifft, M. O.—(1952) *Cancer Res.*, **12**, 707.
Grunberg-Manago, M., Oritz, P. J. and Ochoa, S.—(1955) *Science*, **122**, 907.
Harel, L., Jacob, A. and Moulé, Y.—(1957) *Bull. Soc. Chim. biol., Paris*, **39**, 819.

Hecht, L. L. and Potter, V. R.—(1956) *Cancer Res.*, **16**, 999.
Heidelberger, C.—(1953) *Advanc. Cancer Res.*, **1**, 274.
Herbert, E., Potter, V. R. and Hecht, L. I.—(1957) *J. biol. Chem.*, **225**, 659.
Hurlbert, R. B. and Potter, V. R.—(1954) *Ibid.*, **209**, 1.
Jeener, R. and Szafarz, D.—(1950) *Arch. Biochem.*, **26**, 54.
Keller, E. B., Zamecnik, P. C. and Loftfield, R. B.—(1954) *J. Histochem. Cytochem.*, **2**, 378.
Kuff, E. L. and Hogeboom, G. H.—(1956) ' Enzymes '. Henry Ford Hosp. Symp., ed. O. H. Gaebler. New York (Academic Press), p. 235.
Laird, A. R. and Miller, E. C.—(1953) *Cancer Res.*, **13**, 464.
Leloir, L. F.—(1956) *Conférences et Rapports, 3rd Int. Congr. Biochem.*, Liège (H. Vaillant-Carmanne), p. 154.
Logan, R. and Davidson, J. N.—(1957) *Biochim. Biophys. Acta*, **24**, 196.
Lowe, C. U.—(1955) *J. nat. Cancer Inst.*, **15**, 1619.
Opie, E. L.—(1946) *J. exp. Med.*, **84**, 91.
Petermann, M. L., Mizen, N. A. and Hamilton, M. G.—(1956) *Cancer Res.*, **16**, 620.
Price, J. M., Miller, E. C., Miller, J. A. and Weber, G. M.—(1950) *Ibid.*, **10**, 18.
Reid, E. and Lotz, F.—(1958) *Brit. J. Cancer*, **12**, 419.
*Idem* and O'Neal, M. A.—(1956) *Ibid.*, **10**, 587.
*Idem*, O'Neal, M. A., Stevens, B. M. and Burnop, V. C. E.—(1956) *Biochem. J.*, **64**, 33.
*Idem* and Stevens, B. M.—(1957) *Ibid.*, **67**, 262.—(1958) *Nature*, **182**, 441.
Rutman, R. J., Cantarow, A. and Paschikis, K. E.—(1954) *Cancer Res.*, **14**, 111.
Schneider, W. C., Hogeboom, G. H., Shelton, E. and Striebich, M. J.—(1953) *Ibid.*, 13, 285.
Spain, J. D.—(1956) *Tex. Rep. Biol. Med.*, **14**, 528.
Stevens, B. M. and Reid, E.—(1956) *Biochem. J.*, **64**, 735.
Ward, D. N. and Griffin, A. C.—(1955) *Cancer Res.*, **15**, 456.
Weed, L. L.—(1951) *Ibid.*, **11**, 470.
Zamecnik, P. C., Loftfield, R. B., Stephenson, M. L. and Steele, J. M.—(1951) *Ibid.*, **11**, 592.